# Crisflash: open-source software to generate CRISPR guide RNAs against genomes annotated with individual variation

**DOI:** 10.1093/bioinformatics/btz019

**Published:** 2019-01-12

**Authors:** Adrien L S Jacquin, Duncan T Odom, Margus Lukk

**Affiliations:** 1 Cancer Research UK Cambridge Institute, University of Cambridge, Cambridge, UK and; 2 Cetrale Nantes, 44321 Nantes, Cedex 3, France

## Abstract

**Summary:**

CRISPR/Cas9 system requires short guide RNAs (sgRNAs) to direct genome modification. Most currently available tools for sgRNA design operate only with standard reference genomes, and are best suited for small-scale projects. To address these limitations, we developed Crisflash, a software tool for fast sgRNA design and potential off-target discovery, built for performance and flexibility. Crisflash can rapidly design CRISPR guides against any sequenced genome or genome sequences, and can optimize guide accuracy by incorporating user-supplied variant data. Crisflash is over an order of magnitude faster than comparable tools, even using a single CPU core, and efficiently and robustly scores the potential off-targeting of all possible candidate CRISPR guide oligonucleotides.

**Availability and implementation:**

https://github.com/crisflash

**Supplementary information:**

Supplementary data are available at *Bioinformatics* online.

## 1 Introduction

CRISPR/Cas9 system has become a popular technology for mammalian genome editing, and depends on short, single-stranded guide RNAs (sgRNAs) directing the Cas9 nuclease to a desired locus (Hsu *et al.*, 2013; Ran *et al.*, 2013). The sequence of the sgRNA, typically around 20 bp in length, is fully homologous to its genomic target, followed by a short protospacer adjacent motif (PAM). Current methods design sgRNAs in a desired locus, followed by discovery and scoring of potential off-targets based mainly on their sequence specificity. In the search for potential off-targets for each candidate guide RNA, a score is computed that reflects the total number of fully or partially matching genomic regions (typically capturing up to five mis-matches), as well as the location of mismatches within the candidate guide sequence (Fu *et al.*, 2013).

Most available sgRNA design tools are web-based and created for sgRNA design in a window of up to ten thousand of base pairs (Cradick *et al.*, 2014; Labun *et al.*, 2016; Naito *et al.*, 2014; Singh *et al.*, 2015). Many web-based services have limited support for batch queries, due to computational power required for candidate off-target evaluation, and may provide candidate sgRNAs out of a pool of pre-computed results for a limited set of reference genomes (Hodgkins *et al.*, 2015). To our knowledge, there is no web-based system supporting sgRNA design against custom genome sequences or non-standard genomes. Although some command-line tools are available and mostly free from above limitations (Bae *et al.*, 2014; Xiao *et al.*, 2014; Zhu *et al.*, 2014), a sequence similarity-based search for potential off-targets for thousands to millions of sgRNAs remains a challenge.

Historically, most CRISPR/Cas9-based research has focused on genetic manipulation of human and mouse cell lines and tissues. Accurately generating guide RNAs for gene editing targeted to specific human genomes, or to directly target spontaneous somatic mutations in the human cancer genome requires flexibility to tailor the reference genome and quickly evaluate the quality of candidate sequences. Here, we present Crisflash, a command-line sgRNA design tool built for rapid performance, and flexible and accurate handling of individual genomic variance.

## 2 Materials and methods

Crisflash first scans a FASTA file containing genomic sequences for PAM adjacent protospacers and adds identified gRNAs to an N-ary tree. Nodes correspond to sequence bases; hence, the depth of the tree is equal to the gRNA length. Genomic coordinates of protospacers are saved in terminal nodes. Candidate sgRNAs are then identified, and off-target sequences for these candidate guide RNAs are identified by walking the sequence into the depth of the tree. If a base in a sequence is different than that of a node, a mismatch is recorded, and children of the node are followed as long as the total number of mismatches has not been exceeded. The final score of each candidate sgRNA is computed based on the experimentally observed effects of the mismatch positions of sequences, when followed all the way to the terminal nodes (Hsu *et al.*, 2013). Off-target search and scoring steps for candidates can be run, either in single or multi-threaded mode. For improved design and off-target identification accuracy, Crisflash accepts genetic variation data in vcf format. In case of variant data, after SNP and INDEL information has been incorporated to the genomic sequence, a genome-wide scan for PAMs is executed. For phased variants, two ‘haploid’ genomic sequences are created and gRNAs, as well as modifications in PAM and protospacer areas, are tracked on haplotype level. Creation, deletion and modification of gRNAs in one or both haplotypes is discussed further in Crisflash documentation and in Supplementary Material.

## 3 Results and discussion

**Fig. 1.**
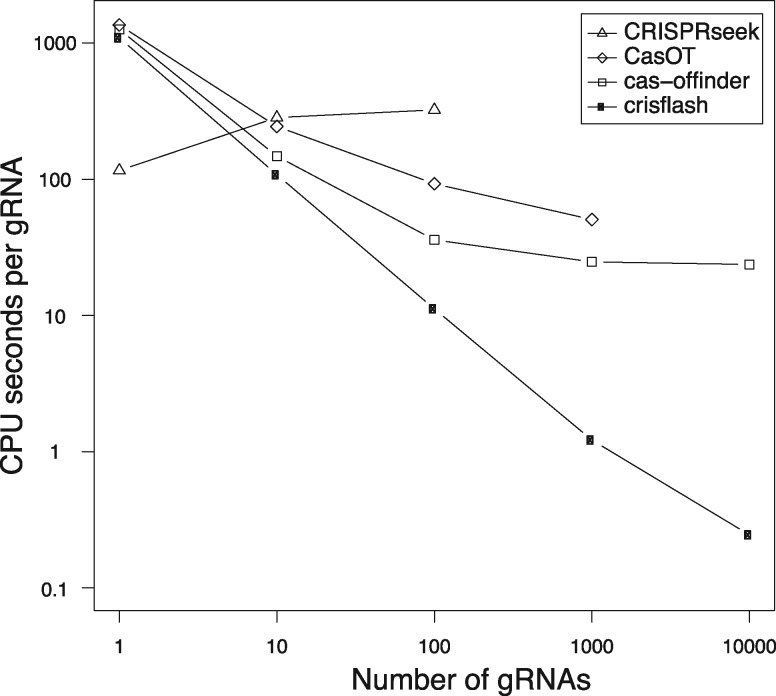
Crisflash performance compared with other command-line sgRNA design tools

We benchmarked Crisflash against following tools: Cas-OFFinder (Bae *et al.*, 2014), CasOT (Xiao *et al.*, 2014) and CRISPRseek (Zhu *et al.*, 2014), all of which are comparable command-line CRISPR design software. All tests were run independently on the same hardware with minimal background computational activity (see Supplementary Material). Off-target search space was limited to four mismatches. In single core mode, Crisflash outperformed all other tools by an order of magnitude for inputs containing more than couple of hundred sgRNAs. The performance is improved above two orders of magnitude for inputs of few thousand candidates compared with cas-offinder while producing similar results (Fig. 1). Tests with higher number of sgRNAs for CasOT and CRISPRseek had to be terminated due to long running times.

The speed advantage gained from tree-based, fast approximate string matching comes with a relatively high requirement for RAM. Existing tools typically rely on a few GB of operational memory. However, the 90 GB necessary for Crisflash to work with human reference genome is readily accommodated in modern server clusters.

Crisflash is first of its kind to offer greater sgRNA accuracy using variant information. Most proposed applications of CRISPR in biomedical research will increasingly demand the flexibility that Crisflash provides in its highly tailored sgRNA design. Crisflash reduces undesirable off-target effects arising from individual human genetic variation and avoids designing non-operational guides.

## 4 Conclusion

Crisflash is a new sgRNA design and potential off-target discovery software, which greatly outperforms similar existing software. Crisflash is built for speed and improved precision when incorporating individual-specific genetic variant data, and is suitable for large-scale guide design, including in non-standard model organisms.

## Funding

This work has been supported by Cancer Research UK core award [20412] and strategic award [22398]. 

*Conflict of Interest*: none declared.

## Supplementary Material

btz019_Supplementary_Methods
