# Supplementary material for: Crisflash: open-source software to generate CRISPR guide RNAs against genomes annotated with individual variation
Source: Bioinformatics. 2019 Jan 12;35(17):3146–7. doi: 10.1093/bioinformatics/btz019 (PMC6735888; doi:10.1093/bioinformatics/btz019)
Supplement: btz019_Supplementary_Methods [file btz019_supplementary_methods.docx]

**Score calculation**

Crisflash score calculation is based on experimentally observed effects of mismatch positions in the target protospacer (Hsu et al., 2013). More specifically, each gRNA is scored by following equation:

$$\boldsymbol{S}_{\boldsymbol{gRNA}}\boldsymbol{=}\frac{\boldsymbol{100}}{\boldsymbol{S}_{\boldsymbol{on-target}}\boldsymbol{+}\sum\boldsymbol{S}_{\boldsymbol{off-target}}}$$

Existing tools (e.g. http://crispr.mit.edu/) simplify the equation by assumption of a single on-target with score of 100:

$$\boldsymbol{S}_{\boldsymbol{gRNA}}\boldsymbol{=}\frac{\boldsymbol{100}}{\boldsymbol{100+}\sum\boldsymbol{S}_{\boldsymbol{off-target}}}$$

The off-target score is computed as follows:

$$\boldsymbol{S}_{\boldsymbol{off-target}}\boldsymbol{=100 \times}\prod_{\boldsymbol{e\in M}} \left( \boldsymbol{1-W}\left[ \boldsymbol{e} \right] \right)\boldsymbol{\times}\frac{\boldsymbol{1}}{\frac{\boldsymbol{19-}\bar{\boldsymbol{d}}}{\boldsymbol{19}}\boldsymbol{\times4+1}}\boldsymbol{\times}\frac{\boldsymbol{1}}{\boldsymbol{n}_{\boldsymbol{mm}}^{\boldsymbol{2}}}$$

M is the length of a list W containing experimentally determined effect scores for each protospacer position

$$\boldsymbol{W}=[0, 0, 0.014, 0, 0, 0.395, 0.317, 0, 0.389, 0.079, 0.445, 0.508, 0.613, 0.851, 0.732, 0.828, 0.615, 0.804, 0.685, 0.583]$$

and d is the mean pairwise distance between mismatches

$$\bar{\boldsymbol{d}}= \frac{pos\max- pos min}{n_{mm}}$$

The equation does not penalize for off-targets with perfect match(es), as the off-target score for sequences with no mismatches is 0. In practice, an exact match against any other sequence than on-target make the candidate highly unattractive. In our implementation we set the off-target score for all perfectly matched off-targets to 100, thus significantly reducing the total score for candidate gRNAs with more than one exact match in the genome.

**Crisflash inputs**

Crisflash inputs for reference genome (option -g) and design target sequence (option -s), are in FASTA format. Option -u is used for limiting gRNA design and genome-wide search to upper case sequences, thus excluding low complexity sequences. Only sequences with standard nucleotide symbols (A,T,G,C,a,t,g,c) are considered. Crisflash memory requirement for human genome with option -u is around ~50GB and ~90GB otherwise.

Running the tool with either -g or -s option, opposed to using both, results in the print-out of all gRNAs with no matching and scoring.

PAM sequence (option -p) may contain any capital IUPAC symbols for nucleotides, consequently making the tool universal to any present and future PAM sequences. For example, PAMs such as NGG, NRG, NNNRYAC, NNAGAW are all valid.

Variant information (option -V) is accepted in VCF format (.vcf suffix expected). Crisflash will proceed if the variant data is not phased. However, we highly recommend phasing the variants first. Using Crisflash with unphased variants may produce incorrect results.

Crisflash supports both SNPs and INDELs and edits the reference sequence accordingly before being used for gRNA discovery. When phased variants are provided, Crisflash will create two improved ‘’haploid genome’’ sequences and will run gRNA discovery for both. Identified gRNAs are marked for their haplotype with possible values being ‘1’, ‘2’ or ‘3’ if gRNA is identical in both haplotypes. Crisflash also records and will report when genomic variation has resulted in any changes to the PAM area (mutation type ‘b’),in protospacer (mutation type ‘c’) or in both (mutation type ‘f’). In the case of no change compared to reference, the mutation type is set to ‘0’.

Applying INDEL data may change the chromosome length and gRNA coordinates; making comparison of gRNA locations difficult. For this reason, all coordinates reported by Crisflash are provided for original reference, even if the gRNA location in variant-adjusted genome may be shifted due to incorporation of INDEL data.

Identification of gRNAs in phased data may result in following three scenarios:

1. gRNAs are identical in both haplotypes – Crisflash reports gRNA with haplotype value ‘3’.
2. gRNAs for the locus are different - we report both gRNAs and label each according to its source haplotype: ‘1’ for variants labelled 1|0 in vcf; and ‘2’ for variants labelled as 0|1. Note: genome-wide search for target and off-target matches for candidates is based on sequence similarity for up to a specified number of mismatches. If one of the gRNAs of the locus (for one of the haplotypes) has more than an expected number of mismatches (specified by option -m), it will not appear in the search results and will not be contributing for scoring. On the other hand, if both gRNAs are matched, the mismatch score will be affected and will be consequently lower. In result, match against the locus with gRNAs differing in haplotypes but those not being too different are penalised by the scoring system.
3. gRNA is present only in one haplotype – This may be due to the deletion or appearance of a new PAM compared with a reference genome in one of the haplotypes. Here, Crisflash reports gRNA for only one haplotype and labels it accordingly.

We suggest careful examination of results for candidates with exact or close to exact matches to only one haplotype.

**Output in BED file format**

By default, Crisflash output is provided in BED file format. In the BED file, the content of the comment filed varies. This depends on whether the program was executed by -g or -s option, or using both. For either -g or -s, the comment filed contains the gRNA sequence followed by ‘:’ and two characters. The first of the two characters is a number (1-3) indicating the haplotype while the other show the mutation type (values ’b’, ’c’, ‘f’, ‘0’).

Executions with -g and -s options result comment field with following information separated by ‘/’: gRNA sequence, the number of of exact matches and the number of approximate matches.

The off-target match score, ranging from 0 to 1, is reported on BED score field. Candidate gRNAs with higher uniqueness have a score closer to 0. Genomic coordinates reported in BED are for the start and end positions of the gRNA in a sequence provided by the option -s. Candidate gRNAs with no exact match have score 0.

**Output in Cas-OFFinder format**

Detailed information about all matching reference sites is provided in Cas-OFFinder format by specifying options -C and -A. Briefly, the first column is for candidate gRNA sequence where the section of PAM sequence is indicated by a sequence of ‘N’. The second and third column records candidate-match positions in reference while the fourth column contains matched-sequence in reference genome with mismatched bases being in lower case. The final two columns are for the chromosome strand and for the number of observed mismatches. Option -A adds an extra column not present in Cas-Offinder tool output. This is for the two-character string, similar to the one already discussed for BED file output: the first of the two characters show the haplotypic origin of the gRNA (values ‘1’ or ‘2’; or ‘3’ if gRNA is identical in both haplotypes); and the second character indicates whether the sequence in PAM area (value ‘b'), protospacer area (value 'c’), both (value 'f') or neither (value ‘0’), was changed by applied variant data.

Crisflash output from option -A needs to be sorted by chromosomes and chromosomal coordinates in order for the gRNAs for both haplotypes (if present and identified by the match) appear on consecutive lines. An example sort command is shown below:

'sort -k1,1 -k2,2 -k3,3 file > file.sorted'

**A comparison of Crisflash and Cas-OFFinder outputs**

Crisflash output in Cas-OFFinder format may not appear identical with output of Cas-OFFinder tool at first glance due to Crisflash output not being sorted by genomic coordinates. After sorting the files by linux sort we find outputs from both tools identical. Example of input files, execution commands for both tools and post-execution sorting of the files is shown below.

Content of crisflash.input.fa:

>chr1:94083642-94083665

TGTGATTGACAAGTGCAGCAAGG

Content of cas-offinder.input:

hg38.fa

NNNNNNNNNNNNNNNNNNNNNGG

TGTGATTGACAAGTGCAGCANNN 4

Cas-OFFinder command:

‘cas-offinder cas-offinder.input C cas-offinder.output’

Crisflash command:

‘crisflash -g hg38.fa -s crisflash.input.fa -o crisflash.output -m 4 -C'

Sorting commands:

‘sort -k1,1 -k2,2 -k3,3 cas-offinder.output > cas-offinder.output.sorted’

‘sort -k1,1 -k2,2 -k3,3 crisflash.output > crisflash.output.sorted’

Comparison of results:

‘diff crisflash.output.sorted cas-offinder.output.sorted’

**Comparison of CRISPR design tools**

Crisflash performance was benchmarked against Cas-OFFinder (version 2.4, opencl version 1.2), CRISPRseek (version 1.20.0, R version 3.5.0) and CasOT (version 1.0). All tools were executed in identical computers with 80 CPU cores and with no background computational activity other than the default processes part of the operating system. All tools were executed in a single code mode with the exception of Cas-OFFinder. Cas-OFFinder cannot be limited to a single processing unit as it is designed to acquire all available compute resource either in CPU or GPU mode. We executed Cas-OFFinder in a CPU mode and observed quick utilization of all 80 CPU cores. Total CPU time used by the programs was calculated by addition of ‘user’ and ‘system’ times reported by Linux command line program ‘time’. All tools were executed by allowing up to 4 mismatches. For CasOT, the number of mismatches was controlled by flag -s, hence limiting all mismatches to 12nt region adjacent to PAM. The reference genome used across all calculations was human hg38 assembly from UCSC.

To minimize bias in measuring tool performance we repeated all benchmark computations 10 times using each time a different set of randomly selected target area. The areas were extended to contain 1, 10, 100, 1000, and 10000 gRNAs (see Table 1 below), allowing up to 10% of additional gRNAs in some cases; and finally, converted the finding from fasta sequences to tool specific input formats of candidate sequences. CPU times reported in Figure 1 represent the average processing times per gRNA across 10 candidate sequence sets. Executions for sets with higher number of gRNAs were completed whenever the tool running time was reasonable. Hence, computations for CRISPRseek and CasOT were were stopped at 100 and 1000 gRNAs respectively.

**Options used for executing the tools**

Crisflash: ‘crisflash -g hg38.fa -s input.fa -o output.bed -m 4’

Cas-OFFinder: ‘cas-offinder input C output’. PAM sequence in the input file was set to ‘NGG’.

CasOT: ‘casot.pl -t input.fa -g hg38.fa -s 4 -p A’

CRISPRseek:

library(CRISPRseek)

library(BSgenome.Hsapiens.UCSC.hg38)

library(TxDb.Hsapiens.UCSC.hg38.knownGene)

results <- offTargetAnalysis(inputFilePath,

findgRNAsWithREcutOnly = FALSE,

findPairedgRNAOnly = FALSE,

BSgenomeName = Hsapiens,

txdb = TxDb.Hsapiens.UCSC.hg38.knownGene,

max.mismatch = 4,

PAM.pattern = 'NGG$',

allowed.mismatch.PAM = 0,

outputDir = outputDir,

overwrite = TRUE)

**Supplementary Table 1**: Genomic coordinates of randomly selected candidate sequences used in benchmarking the tools. The number of gRNAs is calculated for ‘NGG’ PAM sequence.

| Test Set | Nr of gRNAs | Chromosome | Start | End |
| --- | --- | --- | --- | --- |
| 1 | 1 | chr1 | 94,083,642 | 94,083,665 |
| 1 | 10 | chr1 | 94,083,642 | 94,083,800 |
| 1 | 100 | chr1 | 94,083,642 | 94,084,575 |
| 1 | 1,000 | chr1 | 94,083,642 | 94,096,390 |
| 1 | 10,000 | chr1 | 94,083,642 | 94,279,778 |
| 2 | 1 | chr16 | 72,529 | 72,552 |
| 2 | 10 | chr16 | 72,529 | 72,653 |
| 2 | 100 | chr16 | 72,529 | 72,959 |
| 2 | 1,000 | chr16 | 72,529 | 77,502 |
| 2 | 10,000 | chr16 | 72,529 | 164,565 |
| 3 | 1 | chr2 | 127,068,647 | 127,068,670 |
| 3 | 10 | chr2 | 127,068,647 | 127,068,677 |
| 3 | 100 | chr2 | 127,068,647 | 127,068,967 |
| 3 | 1,000 | chr2 | 127,068,647 | 127,073,706 |
| 3 | 10,000 | chr2 | 127,068,647 | 127,164,620 |
| 4 | 1 | chrX | 95,897,654 | 95,897,677 |
| 4 | 10 | chrX | 95,897,654 | 95,898,078 |
| 4 | 100 | chrX | 95,897,654 | 95,901,278 |
| 4 | 1,000 | chrX | 95,897,654 | 95,931,845 |
| 4 | 10,000 | chrX | 95,897,654 | 96,398,290 |
| 5 | 1 | chr15 | 53,408,657 | 53,408,680 |
| 5 | 10 | chr15 | 53,408,657 | 53,408,794 |
| 5 | 100 | chr15 | 53,408,657 | 53,409,517 |
| 5 | 1,000 | chr15 | 53,408,657 | 53,433,044 |
| 5 | 10,000 | chr15 | 53,408,657 | 53,637,877 |
| 6 | 1 | chr8 | 116,359,771 | 116,359,794 |
| 6 | 10 | chr8 | 116,359,771 | 116,360,184 |
| 6 | 100 | chr8 | 116,359,771 | 116,361,560 |
| 6 | 1,000 | chr8 | 116,359,771 | 116,390,408 |
| 6 | 10,000 | chr8 | 116,359,771 | 116,584,472 |
| 7 | 1 | chr2 | 148,622,771 | 148,622,794 |
| 7 | 10 | chr2 | 148,622,771 | 148,622,863 |
| 7 | 100 | chr2 | 148,622,771 | 148,625,931 |
| 7 | 1,000 | chr2 | 148,622,771 | 148,643,444 |
| 7 | 10,000 | chr2 | 148,622,771 | 148,890,612 |
| 8 | 1 | chr6 | 28,732,408 | 28,732,431 |
| 8 | 10 | chr6 | 28,732,408 | 28,732,517 |
| 8 | 100 | chr6 | 28,732,408 | 28,733,479 |
| 8 | 1,000 | chr6 | 28,732,408 | 28,758,022 |
| 8 | 10,000 | chr6 | 28,732,408 | 28,987,010 |
| 9 | 1 | chr2 | 164,400,054 | 164,400,077 |
| 9 | 10 | chr2 | 164,400,054 | 164,400,224 |
| 9 | 100 | chr2 | 164,400,054 | 164,411,502 |
| 9 | 1,000 | chr2 | 164,400,054 | 164,436,354 |
| 9 | 10,000 | chr2 | 164,400,054 | 164,651,468 |
| 10 | 1 | chr18 | 62,565,415 | 62,565,438 |
| 10 | 10 | chr18 | 62,565,415 | 62,565,572 |
| 10 | 100 | chr18 | 62,565,415 | 62,567,525 |
| 10 | 1,000 | chr18 | 62,565,415 | 62,580,560 |
| 10 | 10,000 | chr18 | 62,565,415 | 62,758,506 |
